# Quantum Circuit Architecture Search on a Superconducting Processor

**DOI:** 10.3390/e26121025

**Published:** 2024-11-26

**Authors:** Kehuan Linghu, Yang Qian, Ruixia Wang, Meng-Jun Hu, Zhiyuan Li, Xuegang Li, Huikai Xu, Jingning Zhang, Teng Ma, Peng Zhao, Dong E. Liu, Min-Hsiu Hsieh, Xingyao Wu, Yuxuan Du, Dacheng Tao, Yirong Jin, Haifeng Yu

**Affiliations:** 1 Beijing Academy of Quantum Information Sciences, Beijing 100193, Chinalixg@baqis.ac.cn (X.L.);; 2 School of Computer Science, Faculty of Engineering, University of Sydney, Camperdown, NSW 2006, Australia; 3 JD Explore Academy, Beijing 102628, China; 4 State Key Laboratory of Low Dimensional Quantum Physics, Department of Physics, Tsinghua University, Beijing 100084, China; 5 Centre for Quantum Software and Information, Faculty of Engineering and Information Technology, University of Technology Sydney, Ultimo, NSW 2007, Australia

**Keywords:** quantum architecture search, variational quantum algorithms, noisy intermediate-scale quantum

## Abstract

Variational quantum algorithms (VQAs) have shown strong evidence to gain provable computational advantages in diverse fields such as finance, machine learning, and chemistry. However, the heuristic ansatz exploited in modern VQAs is incapable of balancing the trade-off between expressivity and trainability, which may lead to degraded performance when executed on noisy intermediate-scale quantum (NISQ) machines. To address this issue, here, we demonstrate the first proof-of-principle experiment of applying an efficient automatic ansatz design technique, i.e., quantum architecture search (QAS), to enhance VQAs on an 8-qubit superconducting quantum processor. In particular, we apply QAS to tailor the hardware-efficient ansatz toward classification tasks. Compared with heuristic ansätze, the ansatz designed by QAS improves the test accuracy from 31% to 98%. We further explain this superior performance by visualizing the loss landscape and analyzing effective parameters of all ansätze. Our work provides concrete guidance for developing variable ansätze to tackle various large-scale quantum learning problems with advantages.

## 1. Introduction

The successful exhibition of random quantum circuits sampling and Boson sampling over fifty qubits [1,2,3,4] evidences the potential of using current quantum hardware to address classically challenging problems. A leading strategy towards this goal is variational quantum algorithms (VQAs) [5,6], which leverage classical optimizers to train an *ansatz* that can be implemented on noisy intermediate-scale quantum (NISQ) devices [7]. In the past years, a growing number of theoretical studies have shown the computational superiority of VQAs in the regime of machine learning [8,9,10,11,12,13,14,15,16], quantum many-body physics [17,18,19,20], and quantum information processing [21,22,23]. On par with the achievements, recent studies have recognized some flaws of current VQAs through the lens of the trade-off between expressivity and learning performance [14,24]. That is, an ansatz with very high expressivity may encounter the barren plateau issues [25,26,27,28], while an ansatz with low expressivity could fail to fit the optimal solution [29]. With this regard, designing a problem-specific and hardware-oriented ansatz is of great importance to guarantee the good learning performance of VQAs and the precondition of pursuing quantum advantages.

Pioneered experimental explorations have validated the crucial role of ansatz when applying VQAs to accomplish tasks in different fields such as machine learning [30,31,32,33], quantum chemistry [19,34,35,36,37,38], and combinatorial optimization [39,40,41,42]. On the one side, envisioned by the no-free-lunch theorem [43,44], there does not exist a universal ansatz that can solve all learning tasks with optimal performance. To this end, myriad handcraft ansätze have been designed to address different learning problems [45,46,47]. For instance, the unitary coupled cluster ansatz and its variants attain superior performance in the task of estimating molecular energies [48,49,50,51]. Besides devising the problem-specific ansätze, another indispensable factor to enhance the performance of VQAs is the compatibility between the exploited ansatz and the employed quantum hardware, especially in the NISQ scenario [39]. Concretely, when the circuit layout of ansatz mismatches with the qubit connectivity, additional quantum resources, e.g., SWAP gates, are essential to complete the compilation. Nevertheless, these extra quantum resources may inhibit the performance of VQAs because of the limited coherence time and inevitable gate noise of NISQ machines. Considering that there are countless learning problems and diverse architectures of quantum devices [52,53,54], it is impractical to manually design problem-specific and hardware-oriented ansätze.

To enhance the capability of VQAs, initial studies have been carried out to seek feasible strategies of *automatically designing* a problem-specific and hardware-oriented ansatz with both good trainability and sufficient expressivity. Conceptually, the corresponding proposals exploit random search [55], evolutionary algorithms [56,57], deep learning techniques [58,59,60,61,62,63,64,65], and adaptive strategies [66,67,68] to tailor a hardware-efficient ansatz [19], i.e., inserting or removing gates, to decrease the cost function. In contrast with conventional VQAs that only adjust parameters, optimizing both parameters and circuit layouts enables the enhanced learning performance of VQAs. Meanwhile, the automatic nature endows the power of these approaches to address broad learning problems. Despite the prospects, little is known about the effectiveness of these approaches executed on real quantum devices.

In this study, we demonstrate the first proof-of-principle experiment of applying an efficient automatic ansatz design technique, i.e., quantum architecture search (QAS) scheme [69], to enhance VQAs on an 8-qubit superconducting quantum processor. In particular, we focus on data classification tasks and utilize QAS to pursue a better classification accuracy. To our knowledge, this is the first experimental study of multi-class learning. Moreover, to understand the noise-resilient property of QAS, we fabricate a controllable dephasing noisy channel and integrate it into our quantum processor. Assisted by this technique, we experimentally demonstrate that the ansatz designed by QAS is compatible with the topology of the employed quantum hardware and attains much better performance than the hardware-efficient ansatz [19] when the system noise becomes large. Experimental results indicate that under a certain level of noise, the ansatz designed by QAS achieves the highest test accuracy (95.6%), while other heuristic ansätze only reach 90% accuracy. Additional analyses of loss landscape further explain the advantage of the QAS-based ansatz in both optimization and effective parameter space. These gains in performance suggest the significance of developing QAS and other automatic ansatz design techniques to enhance the learning performance of VQAs.

## 2. Materials and Methods

### 2.1. The Mechanism of QAS

The underlying principle of QAS is optimizing the quantum circuit architecture and the trainable parameters *simultaneously* to minimize an objective function. For elucidating, in the following, we elaborate on how to apply QAS to tailor the hardware-efficient ansatz (HEA). Mathematically, an *N*-qubit HEA $U\left(\theta \right)=\prod_{l=1}^{L}U_{l}\left(\theta \right)\in SU\left(2^{N}\right)$ yields a multi-layer structure, where the circuit layout of all blocks is identical, the *l*-th block $U_{l}\left(\theta \right)$ consists of a sequence of parameterized single-qubit and two-qubits gates, and *L* denotes the block number. Note that our method can be generalized to prune other ansätze, such as the unitary coupled cluster ansatz [49] and the quantum approximate optimization ansatz [70].

**Figure 1:**
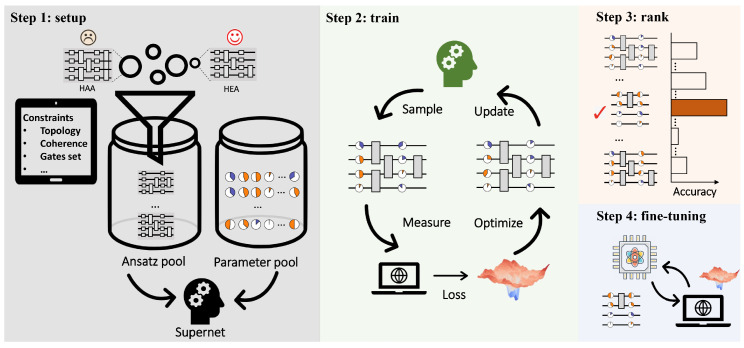
Experimental implementation of QAS. The first step is to construct the ansätze pool S which contains all candidate ansätze satisfying the hardware and physical constraints, such as hardware topology and maximal decoherence time. In the meantime, the parameter pool for all candidate ansätze is initialized in a layer-by-layer manner. The gate arrangement together with corresponding parameters constitutes the supernet. The second step is to sample ansatz from the supernet, measure the observable, calculate the loss, optimize the corresponding parameters based on the objective function, and update the parameters in the supernet. Repeat the above process until reaching the maximal number of iterations. Once the ansatz pool is well trained, the following steps are searching in S, ranking according to performance, and selecting the optimal ansatz for fine-tuning.

QAS is composed of four steps to tailor HEA and output a problem-dependent and hardware-oriented ansatz as shown in Figure 1. The first step is specifying the ansätze pool S, collecting all candidate ansätze. Suppose that $U_{l}\left(\theta \right)$ for ∀l∈[L] can be formed by three types of parameterized single-qubit gates, i.e., rotational gates along three axes, and one type of two-qubits gates, i.e., CNOT gates. When the layout of different blocks can be varied by replacing single-qubit gates or removing two-qubit gates, the ansätze pool S includes in total $O({\left(3^{N}+2^{N}\right)}^{L})$ ansatz. Denote the input data as D and an objective function as L. The goal of QAS is finding the best candidate ansatz a∈S and its corresponding optimal parameters $\theta_{a}^{*}$, i.e.,

(1) $$\left(\theta_{a}^{*},a^{*}\right)=\arg\min_{\theta_{a}\in C,a\in S}L\left(\theta_{a},a,D,E_{a}\right),$$

where the quantum channel $E_{a}$ simulates the quantum system noise induced by a.

The second step is optimizing Equation (1) with, in total, *T* iterations. As discussed in our technical companion paper [69], seeking the optimal solution $\left(\theta_{a}^{*},a^{*}\right)$ is computationally hard since the optimization of a is discrete and the size of S and C exponentially scales with respect to *N* and *L*. To conquer this difficulty, QAS exploits the supernet and weight-sharing strategy to ensure a good estimation of $\left(\theta_{a}^{*},a^{*}\right)$ within a reasonable computational cost. Concisely, the weight-sharing strategy correlates parameters among different ansätze in S to reduce the parameter space C. As for supernet, it plays two significant roles, i.e., configuring the ansätze pool S and parameterizing ansatz a∈S via the specified weight-sharing strategy. In doing so, at each iteration *t*, QAS randomly samples an ansatz $a^{\left(t\right)}\in S$ and updates its parameters, with $\theta_{a}^{\left(t+1\right)}=\theta_{a}^{\left(t\right)}-\eta \nabla L\left(\theta_{a}^{\left(t\right)},a^{\left(t\right)},D,E_{a^{\left(t\right)}}\right)$, and η being the learning rate. Due to the weight-sharing strategy, the parameters of the unsampled ansätze are also updated.

The last two steps are ranking and fine-tuning. Specifically, once the training is completed, QAS ranks a portion of the trained ansätze and chooses the one with the best performance. The ranking strategies are diverse, including random searching and evolutionary searching. Finally, QAS utilizes the selected ansatz to fine-tune the optimized parameters with a few iterations. Refer to Ref. [69] for the omitted technical details of QAS.

### 2.2. Experimental Implementation

We implement QAS on a quantum superconducting processor to accomplish the classification tasks for the Iris dataset. Namely, the Iris dataset $D={\left\{x_{i},y_{i}\right\}}_{i=1}^{150}$ consists of three categories of flowers (i.e., $y_{i}\in \left\{0,1,2\right\}$), and each category includes 50 examples characterized by 4 features (i.e., $x_{i}\in R^{4}$). In our experiments, we split the Iris dataset into three parts, i.e., the training dataset $D_{T}=\left\{x,y\right\}$, the validating dataset $D_{V}$, and the test dataset $D_{E}$ with $D=D_{T}\cup D_{V}\cup D_{E}$. The functionality of $D_{T}$, $D_{V}$, and $D_{E}$ is estimating the optimal classifier, preventing the classifier from being over-fitted, and evaluating the generalization property of the trained classifier, respectively.

**Figure 2:**
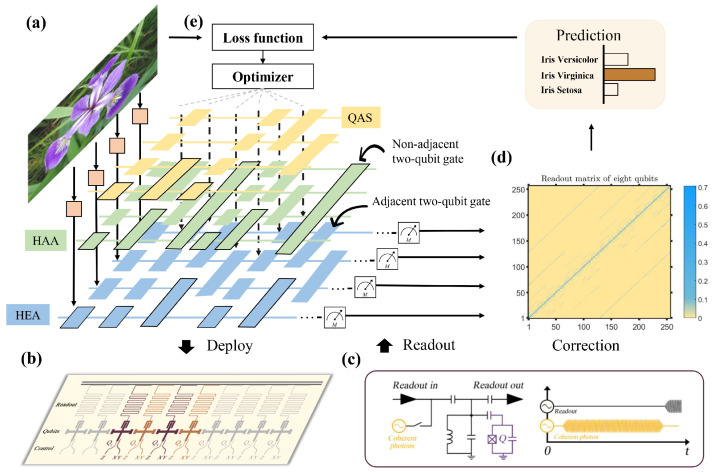
Experimental setups. (**a**) The construction of quantum classifiers with the exploited three different ansätze, i.e., hardware-agnostic ansatz (HAA), hardware-efficient ansatz (HEA), and the ansatz searched by QAS, towards the Iris dataset. For all classifiers, the gate encoding method is adopted to embed the classical feature vector $x_{i}$ into the quantum state $\rho_{i}$. After the interaction of $\rho_{i}$ with the ansatz U(θ), the generated state is measured by a fixed operator Π to obtain the prediction $\mathrm{Tr}(\Pi U\left(\theta \right)\rho_{i}U{\left(\theta \right)}^{†})$. (**b**) All three quantum classifiers are deployed on an 8-qubit superconducting processor with the chain topology. The activated qubits are highlighted by the purple color. (**c**,**d**) To suppress the system noise, error mitigation techniques of measurements are used in our quantum hardware. Namely, the collected measurement results are operated with a correction matrix to estimate the ideal results. Refer to the Method section for details. (**e**) A classical optimizer continuously updates the parameters in U(θ) to minimize the discrepancy between the predictions of quantum classifiers and ground-truth labels indicated by the objective function.

Our experiments are carried out on a quantum processor, including 8 Xmon superconducting qubits with a one-dimensional chain structure. As shown in Figure 2b, the employed quantum device is fabricated by sputtering an aluminum thin film onto a sapphire substrate. The single-qubit rotation gate $R_{X}$ ($R_{Y}$) along X-axis (Y-axis) is implemented with a microwave pulse, and the Z rotation gate $R_{z}$ is realized by virtual Z gate [71]. The construction of the CZ gate is completed by applying the avoided level crossing between the high level states |11〉 and |02〉 or |11〉 and |20〉. The calibrated readout matrix is shown in Figure 2d and the device parameters is summarized in Table A1 of Appendix A.

We fabricate the controllable dephasing noise as a measurable disturbance to the quantum evolution. The operators for the noise channel can be written as $E_{0}=\sqrt{1-\alpha p}\left[1,0;0,1\right]$ and $E_{1}=\sqrt{\alpha p}\left[1,0;0,-1\right]$. α is a constant, and the value of *p* can be tuned in our experiment by changing the average number of the coherent photons on the readout cavity’s steady state. The intensity of coherent photons is represented by the amplitude *p* of the curve shown on the AWGs.

The experimental implementation of the quantum classifiers is as follows. As illustrated in Figure 2a, the gate encoding method is exploited to load classical data into quantum states. The encoding circuit yields $U_{E}\left(x\right)={⊗}_{j=1}^{4}R_{Y}\left(x_{i,j}\right)$. To evaluate the effectiveness of QAS, three types of ansätze U(θ) are used to construct the quantum classifier. The first two types are heuristic ansätze, which are the hardware-agnostic ansatz (HAA) and hardware-efficient ansatz (HEA). As depicted in Figure 2a, HAA $U_{\mathrm{HAA}}\left(\theta \right)$ is designed for a general paradigm and ignores the topology of specific quantum hardware platforms; HEA $U_{\mathrm{HEA}}\left(\theta \right)$ adapts to the quantum hardware constraints, where all inefficient two-qubit operators that connect two physically nonadjacent qubits are forbidden. The third type of ansatz refers to the output of QAS, denoted as $U_{\mathrm{QAS}}\left(\theta \right)$. The mean square error between the prediction and real labels is employed as the objective function for all quantum classifiers. The noise rate of the dephasing channel *p* is set as 0, 0.01, and 0.015. We benchmark the test accuracy of these three ansätze HAA, HEA, and QAS, and explore whether QAS attains the highest test accuracy. Refer to Appendix B for more implementation details.

## 3. Results

**Figure 3:**
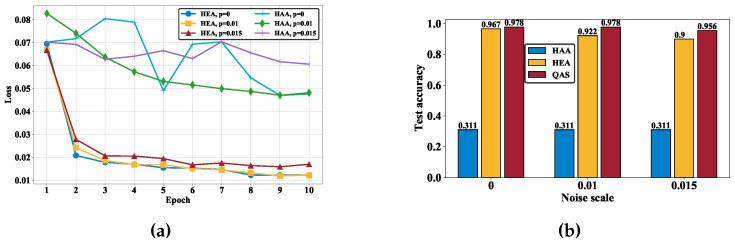
The performance of quantum classifiers. (**a**) The training loss of quantum classifiers with the HAA and HEA ansätze under different noise settings. (**b**) The test accuracy achieved by HAA, HEA, and the ansatz searched by QAS under different noise settings.

To comprehend the importance of the compatibility between quantum hardware and ansätze, we first examine the learning performance of the quantum classifiers with HAA and HEA under different noise rates. The achieved experimental results are demonstrated in Figure 3a. In particular, in the measure of training loss (i.e., the lower the better), the quantum classifier with the HEA significantly outperforms HAA for all noise settings. At the 10-th epoch, the training loss of the quantum classifier with HAA and HEA is 0.06 and 0.017 (0.049 and 0.012; 0.049 and 0.012) when p=0.015 (p=0; p=0.01), respectively. In addition, the optimization of the quantum classifier with HAA seems to be divergent when p=0.015. We further evaluate the test accuracy to compare their learning performance. As shown in Figure 3b, there exists a manifest gap between the two ansätze, highlighted by the blue and yellow colors. For all noise settings, the test accuracy corresponding to HAA is only 31.1%, whereas the test accuracy corresponding to HEA is at least 95.6%. These observations signify the significance of reconciling the topology between the employed quantum hardware and ansatz as the key motivation of this study. Specifically, the performance gap between HAA and HEA drives us to introduce QAS, which focuses on searching for the optimal quantum circuit architecture rather than iteratively updating gate parameters within a single, fixed architecture.

We next experiment on QAS to quantify how it is a problem-specific and hardware-oriented design to enhance the learning performance of quantum classifiers. Concretely, as shown in Figure 3b, for all noise settings, the quantum classifier with the ansatz searched by QAS attains the best test accuracy compared to those of HAA and HEA. That is, when p=0 (p=0.01 and p=0.015), the test accuracy achieved by QAS is 97.8% (97.8% and 95.6%), which is higher than HEA with 96.7% (92.2% and 90.0%). Notably, although the test accuracy is slightly decreased for the increased system noise, the strength of QAS becomes evident over the other two ansätze. In other words, QAS shows the advantages of simultaneously alleviating the effect of quantum noise and searching the optimal ansatz to achieve high accuracy. The superior performance validates the effectiveness of QAS in classification tasks.

**Figure 4:**
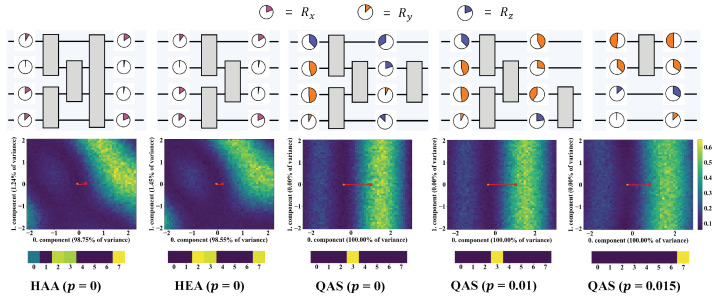
The circuit architecture and corresponding loss landscape. The top row demonstrates the structure of HAA, HEA, and the ansatz searched by QAS under different noise settings. The color and angles refer to the gate type and corresponding parameter. The middle row visualizes the loss landscape of each ansatz with respect to the trained parameters based on the technique developed in [73]. The red line tracks the optimization path of loss during the 50 epochs. The linear path indicates that the loss landscape enjoys a simple structure and optimization is easy to converge. The bottom row shows the absolute value of the first row vector of the PCA transform matrix, which reflects the contribution of each parameter to the first component. Numbers 0–7 denote the parameter index.

We last investigate the potential factors of ensuring the good performance of QAS from two perspectives, i.e., the circuit architecture and the corresponding loss landscape. The searched ansätze under three noise settings, HAA, and HEA are pictured at the top of Figure 4. Compared with HEA and HAA, QAS reduces the number of CZ gates with respect to the increased level of noise. When p=0.015, QAS chooses the ansatz containing only one CZ gate. This behavior indicates that QAS can adaptively control the number of quantum gates to balance the expressivity and learning performance. We plot the loss landscape of HAA, HEA, and the ansatz searched by QAS in the middle row of Figure 4. To visualize the high-dimension loss landscape in a 2D plane, the dimension reduction technique, i.e., principal component analysis (PCA) [72] is applied to compress the parameter trajectory corresponding to each optimization step. After dimension reduction, we choose the obtained first two principal components that explain most of the variance as the landscape spanning vector. Refer to [73] and Appendix C for details. For HAA and HEA, the objective function is governed by both the 0-th component (98.75% of variance for HAA, 98.55% of variance for HEA) and 1-th component (1.24% of variance for HAA, 1.45% of variance for HEA). By contrast, for the ansätze searched by QAS, their loss landscapes totally depend on the 0-th component. Furthermore, the optimization path for QAS is exactly linear, while the optimization of HAA and HAA experiences a nonlinear curve. This difference reveals that QAS enables a more efficient optimization trajectory. As indicated in the bottom row of Figure 4, there is a major parameter that contributes the most to the 0-th component in the three ansätze searched by QAS, while HAA and HEA have to consider multiple parameters to determine the 0-th component. This phenomenon reflects that ansätze searched by QAS are prone to having a smaller effective parameter space, which leads to less noise accumulation and further stronger noise robustness. These observations can be treated as empirical evidence to explain the superiority of QAS.

## 4. Discussion

Our experimental results provide the following insights. First, we experimentally verify the feasibility of applying automatically designing a problem-specific and hardware-oriented ansatz to improve the power of quantum classifiers. Second, the analysis related to the loss landscape and the circuit architectures exhibits the potential of applying QAS and other variable ansatz construction techniques to compensate for the caveats incurred by executing variational quantum algorithms on NISQ machines.

Besides classification tasks, it is crucial to benchmark QAS and its variants towards other learning problems in quantum chemistry and quantum many-body physics. In these two areas, the employed ansatz is generally Hamiltonian dependent [48,49,74]. As a result, the way of constructing the ansatz pool should be carefully conceived. In addition, another important research diction is understanding the capabilities of QAS for large-scale problems. How to find the near-optimal ansatz among the exponential candidates is a challenging issue.

We note that although QAS can reconcile the imperfection of quantum systems, a central law to enhance the performance of variational quantum algorithms is promoting the quality of quantum processors. For this purpose, a promising area for future exploration is to delve into carrying out QAS and its variants on more types of quantum machines to accomplish more real-world generation tasks with potential advantages.

## Figures and Tables

**Figure A1:**
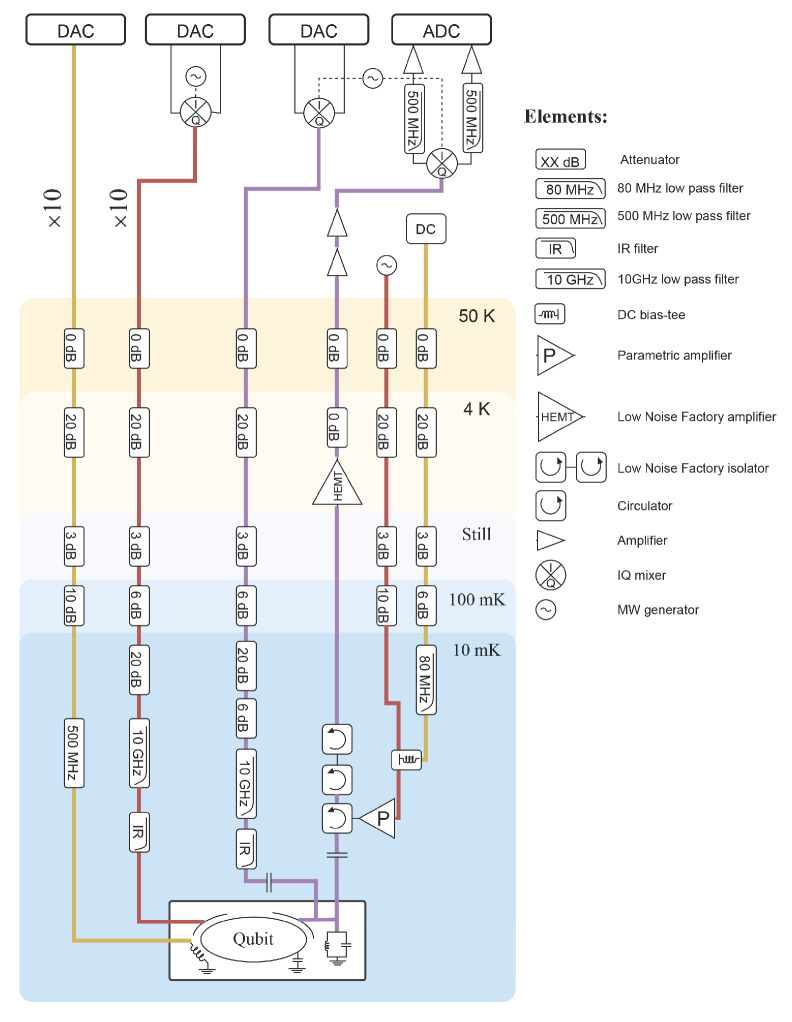
Schematic diagram for the electronics and wiring setup for the superconducting quantum system.

**Figure A2:**
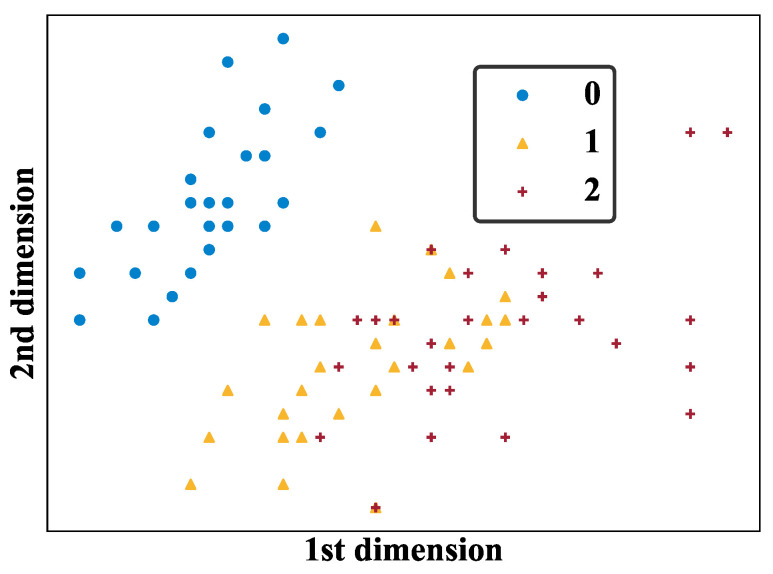
The visualization of the Iris dataset based on its first two dimensions.

**Figure A3:**
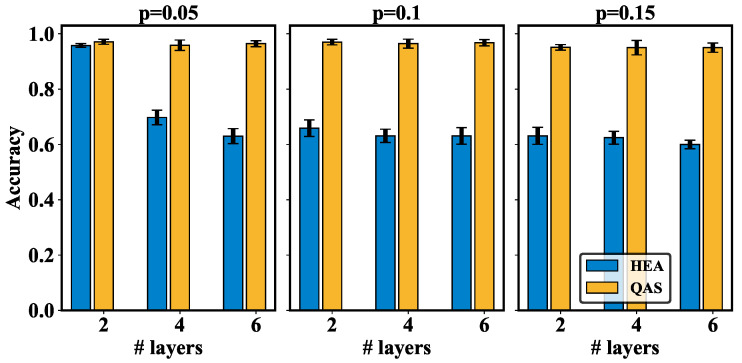
The test accuracy achieved by HEA and QAS under various numbers of layers and noise strength when simulating on classical devices.

**Figure A4:**
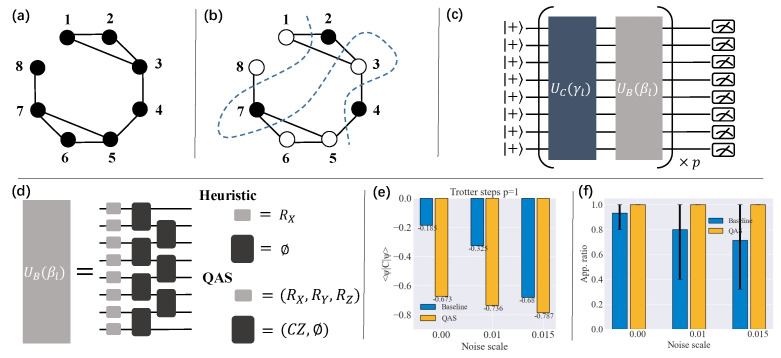
Experimental results for the QAOA. (**a**) An instance of a graph with 8 vertices. (**b**) An optional partition achieving the max cut. (**c**) The QAOA circuit. (**d**) QAS helps design $U_{B}$. (**e**) The loss comparison between standard QAOA (termed ‘baseline’) and QAS. (**f**) The approximation ratio achieved by standard QAOA and QAS.

## Data Availability

Data will be shared when there is a demand.

## Appendix A. Experiment Setup

### Appendix A.1. Device Parameters

The qubit parameters, length, and fidelity for the single- and two-qubit gates of our device are summarized in Table A1.

### Appendix A.2. Electronics and Control Wiring

The device is installed in a cryogenic setup in a dilution refrigerator. The control and measurement electronics which are connected to the device are shown in Figure A1. The electronic control module is divided into six areas with different temperatures. The superconducting quantum device is installed at the base plate with a cryogenic environment of 10 mK. For each qubit, the frequency is tuned by a flux control line by changing the magnetic flux through the SQUID loop, and the flux is controlled by a constant current, which is generated by a voltage source and inductively coupled to the SQUID. Four attenuators are connected in the circuit in series to act as the thermal precipitator. The XY control for each qubit is achieved by up-converting the intermediate frequency signals with an analog IQ-mixer module. The drive pulse is provided by the multi-channel AWGs with a sample rate of 2 GSa/s.

**Table A1:** Device parameters. $\omega_{i}$ and $\alpha_{i}$ represent the qubit frequency and qubit anharmonicity, respectively. $T_{1}$ and $T_{2}^{★}$ are the longitudinal and transverse relaxation times, respectively. ${\bar{F}}_{s}$ and $T_{s}$ are the average fidelity and length of the single-qubit gates. $g_{i}$ is the coupling strength between nearby qubits, and $J_{z,i}$ is the effective ZZ coupling strength. ${\bar{F}}_{cz}$ are the fidelity of the CZ gates calibrated by quantum process tomography, and $T_{cz}$ are the length of the CZ gates.

Parameter	Q1		Q2		Q3		Q4		Q5		Q6		Q7		Q8
$\omega_{i}/2\pi$ (GHz)	5.202		4.573		5.146		4.527		5.099		4.47		5.118		4.543
$\alpha_{i}/2\pi$ (GHz)	−0.240		−0.240		−0.239		−0.240		−0.239		−0.239		−0.239		−0.242
$T_{1}$ (μs)	10.3		13.6		14.7		16.7		14.9		13.2		12.7		8.0
$T_{2}^{★}$ (μs)	11.7		2.0		15.2		1.9		15.9		1.8		12.6		1.6
${\bar{F}}_{s}$	99.67%		98.96%		99.76%		99.55%		99.05%		99.69%		98.51%		99.09%
$T_{s}$ (ns)	37		37		37		35		35		37		37		35
$g_{i}/2\pi \;\left(\mathrm{MHz}\right)$		19.7		19.6		19.1		19.1		19.2		19.3		19.6	
$J_{z,i}/2\pi \;\left(\mathrm{MHz}\right)$		0.675		0.8		0.7		0.78		0.626		0.655		0.819	
${\bar{F}}_{cz}$		97.23%		96.11%		97.48%		93.54%		93.72%		94.95%		95.54%	
$T_{cz}$ (ns)		18.5		21.5		23		19.5		23.5		21.5		18.5	

The qubit readout is performed by a readout control system with a sampling rate of 1 GSa/s. The readout pulse is up-converted to the frequency band of the readout cavity with the analog IQ-mixer and transmitted through the readout line with attenuators and low-pass filters to the chip. At the output side, the constant and alternating currents are combined by a bias-tee, amplified by the parametric amplifier, and connected to the readout line through a circulator at 10 mK, as well as a high-electron-mobility transistor (HEMT) at 4 K and two more amplifiers at room temperature. The amplified signals finally are digitized by an analog-to-digital converter.

### Appendix A.3. Noise Setup

Due to the ac Stark effect, photon number fluctuations from the readout cavity can cause qubit dephasing [75]. We implement a pure dephasing noisy channel in our device. To every qubit, the noise photons are generated by a coherent source with a Lorentzian-shaped spectrum, which is centered at the frequency of $\omega_{c}$. $\omega_{c}$ is the center frequency of the readout cavity, which is over-coupled to the feedline at the input and output port, and capacitively coupled to the Xmon qubit. The Hamiltonian system including the readout cavity and the qubit can be written as

(A1) $$H/\hbar =\omega_{c}a^{†}a+\frac{\omega_{q}}{2}\sigma_{z}+g_{r}\left(a^{†}\sigma_{-}+a\sigma_{+}\right)$$

where $\sigma_{\pm }=\sigma_{x}\pm i\sigma_{y}$, and $\sigma_{j}$ (j=x,y,z) is the Pauli operator for the X-mon qubit. $a^{†}$ (*a*) is the cavity photon creation (annihilation) operator. $\omega_{q}$ is the frequency between the ground and the first excited states of the qubit, and $g_{r}$ is the coupling strength between the qubit and the readout cavity.

By continuously sending the coherent photons to drive the readout cavity to maintain a coherent state, a noisy environment can be engineered. The noise channel can be described as the depolarization in the x-y plane of the Bloch sphere. The noise intensity can be tuned by changing the average number of coherent photons on the readout cavity’s steady state. The average number of photons is represented by the amplitude of the curve shown in the AWGs. Under different noise settings, the values of $T_{2}^{★}$ are shown in Table A2.

**Table A2:** The transverse relaxation time $T_{2}^{★}$ under different noise settings.

Parameter	Q1	Q2	Q3	Q4	Q5	Q6	Q7	Q8
$T_{2}^{★}\left(p=0\right)$ (μs)	11.7	2.0	15.2	1.9	15.9	1.8	12.6	1.6
$T_{2}^{★}\left(p=0.005\right)$ (μs)	10.6	1.9	15.3	1.8	15.0	1.6	10.7	1.5
$T_{2}^{★}\left(p=0.01\right)$ (μs)	7.8	1.6	12.5	1.7	14.7	1.6	13.0	1.6
$T_{2}^{★}\left(p=0.015\right)$ (μs)	0.2	1.2	12.7	1.6	14.4	1.6	12.1	1.5

### Appendix A.4. Readout Correction

The experimentally measured results of the final state for the eight qubits were corrected with a calibration matrix, which can be obtained in an experimental calibration process. The reconstruction process for readout results is based on Bayes’ rule. The colored schematic diagram for the calibration matrix is shown in Figure 2d. Assume that $p_{ij}$ stands for the probability of obtaining a measured population |i〉 when preparing a basis state |j〉. The calibration matrix is

(A2) $$\begin{array}{c} F=\left(\begin{array}{cccc} p_{11} & p_{12} & ... & p_{12^{n}}\\ p_{21} & p_{22} & ... & p_{22^{n}}\\ ... & ... & ... & ...\\ p_{2^{n}1} & p_{2^{n}2} & ... & p_{2^{n}2^{n}} \end{array}\right). \end{array}$$

If we prepare a state |ψ〉 on *n* qubits, and the probability distribution of the prepared state in $2^{n}$ basis is $P={\left[P_{1},P_{2},...,P_{2^{n}}\right]}^{T}$, then we will obtain a measured state probability distribution as $\tilde{P}$ in the experiment. The relationship between the two probability distributions is

(A3) $$\tilde{P}=FP.$$

Sovling for *P*, we have

(A4) $$P=F^{-1}\tilde{P}.$$

## Appendix B. Implementation of Quantum Classifiers

In this section, we implement HAA, HEA, and QAS for the classification of the Iris dataset on the 8-qubit superconducting quantum processors with controllable dephasing noise. A detailed description of the dataset and hyper-parameters configuration is given below.

### Appendix B.1. Dataset

The classification data employed in this paper are from the Iris dataset [76], which contains 150 instances characterized by 4 attributes and 3 categories. Each dimension of the feature vector is normalized to range [0,1]. During training, the whole dataset is split into three parts, including the training set (60 samples), validation set (45 samples), and test set (45 samples). The sample distribution is visualized by selecting the first two dimensions of the feature vector. As shown in Figure A2, samples of class 1 and 2 cannot be distinguished by a linear classifier. It means that nonlinearity should be introduced into the quantum classifier to achieve higher classification accuracy.

### Appendix B.2. Objective Function and Accuracy Measure

Objective function. We adopt the mean square error (MSE) as the objective function for all quantum classifiers, i.e.,

(A5) $$L\left(D,\theta \right)=\frac{1}{2n}\sum_{i=1}^{n}{\left({\langle O\rangle }_{i}-y_{i}\right)}^{2},$$

where ${\langle O\rangle }_{i}=\langle 0|U_{E}{\left(x_{i}\right)}^{†}U{\left(\theta \right)}^{†}OU\left(\theta \right)U_{E}\left(x_{i}\right)|0\rangle$, *O* refers to the observable, $U_{E}\left(x_{i}\right)$ denotes the unitary operator that embeds classical feature vector $x_{i}$ into the quantum circuit, and U(θ) is the variational quantum circuit with the trainable parameters θ.

Definition of train, valid, and test accuracy. Given an example $x_{i}$, the quantum classifier predicts its label as

(A6) $${\tilde{y}}_{i}=\left\{\begin{array}{cc} 0, & {\langle O\rangle }_{i}\leq \frac{1}{6}\\ 1, & \frac{1}{6}<{\langle O\rangle }_{i}\leq \frac{1}{2}\\ 2, & \frac{1}{2}<{\langle O\rangle }_{i}\leq \frac{5}{6} \end{array}\right..$$

The train (valid and test) accuracy aims to measure the differences between the predicted labels and true labels for examples in the training dataset $D_{T}$ (valid dataset $D_{V}$ and test dataset $D_{E}$), i.e., 3,

(A7) $$accuracy=\frac{\sum_{(x_{i},y_{i})\in D}1_{{\tilde{y}}_{i}=y_{i}}}{\left|D\right|},D=D_{T}\;or\;D_{V}\;or\;D_{E},$$

where |·| denotes the size of a set.

### Appendix B.3. Training Hyper-Parameters

The trainable parameters for all ansätze are randomly initialized following the uniform distribution $U_{\left[-\pi ,\pi \right]}$. During training, the hyper-parameters are set as follows: the optimizer is stochastic gradient descent (SGD) [77], the batch size is 4, and the learning rate is fixed at 0.2. Specifically, the parameter shift rule [78] is applied to compute the gradient of the objective function with respect to a single parameter.

For QAS, we train 5 candidate supernets for 40 epochs to fit the training set. During the search phase, we randomly sample 100 ansatz and rank them according to their accuracy on the validation set. Finally, the ansatz with the highest accuracy is selected as the target ansatz. The ansätze pool is constructed as follows. For the single-qubit gate, the candidate set is {RY,RZ}. For the two-qubit gate, QAS automatically determines whether to apply CZ gates to the qubit pair (0,1),(1,2),(2,3) or not, discarding all other combinations, such as (0,2) and (0,3). These nonadjacent qubits connections require more gates when running on the superconducting processor of 1-D chain topology, leading to bigger noise accumulation.

## Appendix C. More Details of the Results

### Appendix C.1. PCA Used in the Visualization of Loss Landscape

To visualize the loss landscape of HAA, HEA, and ansätze searched by QAS with respect to the parameter space, we apply principle component analysis (PCA) to the parameter trajectory collected in every optimization step and choose the first two components as the observation variable. To be concrete, given a sequence of trainable parameter vectors along the optimization trajectory $\left\{\theta^{\left(1\right)},...,\theta^{\left(t\right)},...,\theta^{\left(T\right)}\right\}$, where *T* is the number of total optimization steps and $\theta^{\left(t\right)}\in R^{d}$ denotes the parameter vector at the *t*-th step, we construct the matrix $\Theta =\left[\theta^{\left(1\right)};...;\theta^{\left(T\right)}\right]\in R^{T\times d}$. Once we apply PCA to Θ and obtain the first two principal components $E={\left[e_{0},e_{1}\right]}^{T}\in R^{2\times d}$, the loss landscape with respect to trainable parameters can be visualized by performing a 2D scan for $E\Theta^{T}$. Simultaneously, the projection vector $e_{i}$ of each component indicates the contribution of each parameter to this component, implying how many parameters determine the value of the objective function. Refer to [73] for details.

The optimization trajectory can provide certain information on the trainability and convergence of the employed ansatz in quantum classifiers. When the optimization path is exactly linear, it implies that the loss landscape is not intricate and the model can be easily optimized. On the contrary, the complicated nonlinear optimization curve indicates the difficulty of covering to the local minima.

### Appendix C.2. Simulation Results

We conduct numerical experiments on classical computers to validate the effectiveness of QAS.

Dephasing noise. We simulate the dephasing noise channel as

(A8) $$\rho^{\prime }=\left(1-\bar{p}\right)\rho +\bar{p}\sigma_{z}\rho \sigma_{z},$$

where ρ and $\rho^{\prime }$ represent the ideal quantum state (density matrix) and noisy quantum state affected by the dephasing channel, $\sigma_{z}$ is the Pauli-Z operator, and $\bar{p}=\alpha p$ is the noise strength, representing the probability of applying a Pauli-Z operator to the quantum state. In the experiments, the noise strength $\bar{p}$ is set as {0.05,0.1,0.15}, and the circuit layer *L* is set as {2,4,6}. Each setting runs 10 times to suppress the effects of randomness.

Simulation results. As shown in Figure A3, QAS achieves the highest test accuracy over all noise and layer settings. When L=2 and p=0.05, the performance gap between HEA (95.7%) and QAS (97.1%) is relatively small. With both the depth and noise strength increasing, HEA witnesses a rapid accuracy drop (60% for L=6 and p=0.15). By contrast, the test accuracy for QAS with L=6 and p=0.15 is 95%, which slightly decreases 2%. This behavior accords with the results on the superconducting processor (the test accuracy of QAS running a superconducting device decreases from 97.8% to 95.6% when *p* increases from 0 to 0.015; refer to Figure 3 for more details), further illustrating the advantage of QAS in error mitigation and model expressivity.

### Appendix C.3. QAS in Quantum Approximate Optimization Algorithm (QAOA)

The quantum approximate optimization algorithm [41] is a variational quantum algorithm used to solve combinatorial optimization problems. In our work, QAOA is employed to search the maximum cut of an undirected graph. To this end, the system Hamiltonian generated from a graph G=(V,E) with vertices *V* and edges *E* is given as

(A9) $$C=\sum_{j,k\in E}Z_{j}Z_{k},$$

where $Z_{j}$ is the Pauli-Z operator. Theoretically, the optimal partition ψ is exactly deduced from the state |ψ〉, minimizing the expectation 〈ψ|C|ψ〉. In practice, the target state is approximated by following variation quantum circuit with depth *p*:

(A10) $$|\gamma ,\beta \rangle =\prod_{l=1}^{p}U_{B}\left(\beta_{l}\right)U_{C}\left(\gamma_{l}\right)|+\rangle ,$$

where the unitary gate $U_{C}\left(\gamma_{l}\right)$ is generated by the Hamiltonian $U_{C}\left(\gamma_{l}\right)=\exp\left(-i\gamma_{l}C\right)$, $U_{B}\left(\beta_{l}\right)$ is usually selected as $\exp\left(-i\beta_{l}X\right)$, γ, and β are trainable parameters. Following the hybrid quantum–classical optimization scheme, we can asymptotically approach the solution by minimizing the expectation 〈γ,β|C|γ,β〉 with infinite *p*.

Considering the system noise and limited qubit connectivity of quantum hardware, a natural generalization of $U_{B}$ is a generic unitary operator with free rotation axis [79] or more trainable parameters [80,81,82]. Inspired by the success of QAS in classification, we also apply QAS to automatically search the optimal mixer Hamiltonian to match the qubit connectivity and mitigate error.

We study the 8-qubit system for the max-cut problem of graph with 8 vertices. Due to the chain connection of physical qubits, edges connecting vertices $V_{i}$ and $V_{j}$ with |i−j|>2 are discarded as shown in Figure A4a. The graph node indexed from 1 to 8 corresponds to the qubits from Q3 to Q10. An optional graph partition achieving the maximum cut 7 is depicted in Figure A4b. Following the protocol of QAOA and the target graph, we employ the ansatz in Figure A4c to search the max-cut, where $U_{C}$ is generated by the system Hamiltonian *C*, and $U_{B}$ is generated by the mixer Hamiltonian. As drawn in Figure A4d, the heuristic layout of $U_{B}$ is described by $U_{B}\left(\beta_{l}\right)=R_{X}{\left(\beta_{l}\right)}^{⊗8}$, while QAS introduces more candidate Pauli operators and entangling gates to enhance the flexibility of $U_{B}$. The performance of QAS for QAOA is demonstrated in Figure A4e,f. It is obvious that the mixer Hamiltonian searched by QAS achieves smaller loss 〈ψ|C|ψ〉 than the vanilla mixer Hamiltonian for various trotter steps and noise scales, except for the extreme setting of p=2 and 0.015 noise strength. We further measure the approximation ratio $r=\frac{A}{A_{max}}$, where *A* is the graph cut solved by QAOA, and $A_{max}$ is the theoretical maximum cut. When corrupted by noise with increasing scales, the standard QAOA witnesses a gradual reduction in the average approximation ratio. Instead, the quantum ansatz generated by QAS can guarantee to find the optimal solution even when suffering from noise of intensity 0.015.
